# Supplementary material for: Susceptibility toward cefiderocol and sulbactam-durlobactam in extensively drug-resistant Acinetobacter baumannii detected from ICU admission screening in Hanoi, Vietnam, 2023
Source: Microbiol Spectr. 2025 Jun 9;13(7):e00832-25. doi: 10.1128/spectrum.00832-25 (PMC12210873; doi:10.1128/spectrum.00832-25)
Supplement: Supplemental material — Table S1, and Fig. S1 and S2. [file spectrum.00832-25-s0002.docx]

**Susceptibility towards cefiderocol and sulbactam-durlobactam in extensively drug-resistant *Acinetobacter* *baumannii* detected from ICU admission screening in Hanoi, Vietnam, 2023**

**Authors:**

Sébastien Boutin ^1,2,3, *^, Nguyen Quang Toan^4,5,*^, Thi Anh Mai Pham^1,4^, Truong Nhat My^4^, Nguyen Thi Kim Phuong^5^, Bui Tien Sy^4,6^, Nguyen Van Trong ^6^, Lisa Göpel^1^, Leo Huber^1^, Kaan Kocer^1^, Le Thi Kieu Linh, Tran Thanh Tung^1^, Nguyen Trong The^4,7^,, Le Huu Song^4,7^, Thirumalaisamy P. Velavan^4,8,9^, Dennis Nurjadi^1,2,4^

* equal contributions

**Affiliations:**

^1^ Institute of Medical Microbiology and Center for Infectious Diseases, University of Lübeck and University Medical Center of Schleswig-Holstein Campus Lübeck, Lübeck, Germany.

^2^German Center for Infection Research (DZIF), Partner Site Hamburg-Lübeck-Borstel-Riems, Hamburg-Lübeck-Borstel-Riems, Lübeck, Germany.

^3^Airway Research Center North (ARCN), Lübeck, Germany, Member of the German Center for Lung Research (DZL)

^4^Vietnamese - German Centre for Medical Research (VG-CARE), Hanoi, Vietnam.

^5^Department of Infection Control, 108 Military Central Hospital, Hanoi, Vietnam.

^6^Department of Microbiology, 108 Military Central Hospital, Hanoi, Vietnam.

^7^Department of Infectious Diseases, 108 Military Central Hospital, Hanoi, Vietnam.

^8^Institute of Tropical Medicine, University of Tübingen, Wilhelmstrasse 27, 72074, Tübingen, Germany.

^9^Faculty of Medicine, Duy Tan University, Da Nang, Vietnam

**Supplementary Table 1. Demographic and clinical characteristics of Carbapenem resistant *A. baumannii* colonization stratified by colonization/infection.** Complete clinical data was only available for 28 of 31 patients colonized with CRAB. Abbreviations: CRAB=carbapenem-resistant *A. baumannii*, M=male, F=female, MLST=multi-locus sequence type

|  | **Total, n=31** | |  |
| --- | --- | --- | --- |
|  |  |  |  |
|  | n | % |  |
| **basic demographics** |  |  |  |
| Age, median (IQR) | 68 | (55.5-79) |  |
| female sex | 8 | 28.6 |  |
| length of stay (median, IQR) | 13 | 7.5-24.5 |  |
|  |  |  |  |
| **Time of first positivity** |  |  |  |
| admission | 8 | 25.8 |  |
| during | 18 | 58.1 |  |
| discharge | 3 | 9.7 |  |
|  |  |  |  |
| **Clinical outcome** |  |  |  |
| discharge | 9 | 29.0 |  |
| Transfer to non-ICU unit | 13 | 41.9 |  |
| dead | 6 | 19.4 |  |
|  |  |  |  |
| **Antibiotic resistance** |  |  |  |
| Imipenem | 31 | 100 |  |
| Meropenem | 31 | 100 |  |
| Ciprofloxacin | 31 | 100 |  |
| Levofloxacin | 31 | 100 |  |
| Trimethoprim-sulfamethoxazole | 28 | 90.3 |  |
| Amikacin | 30 | 96.8 |  |
| Colistin | 2 | 6.5 |  |
| Cefiderocol | 4 | 12.9 |  |
| Sulbactam-Durlobactam | 7 | 22.6 |  |
|  |  |  |  |
| **MLST** |  |  |  |
| ST2 | 18 | 64.3 |  |
| ST16 | 3 | 0.1 |  |
| ST164 | 4 | 0.1 |  |
| Others | 3 | 0.1 |  |

**Supplementary Dataset S1. Sequencing data, metadata and antimicrobial susceptibility**

The excel file contains all the sequencing quality data, metadata and antimicrobial susceptibility testing, the antimicrobial resistance and virulence gene found in the dataset. The SNPs/Indel/Insertion file is available upon request due to file size limitation for supplementary datasets.

**Supplementary figure 1.** Minimum Spanning Tree based on the SNP distance corrected for recombination using Gubbins. The algorithm used for the MST was MSTree v2 and edges < 10 were concatenated. MLST was defined by the Pasteur scheme.


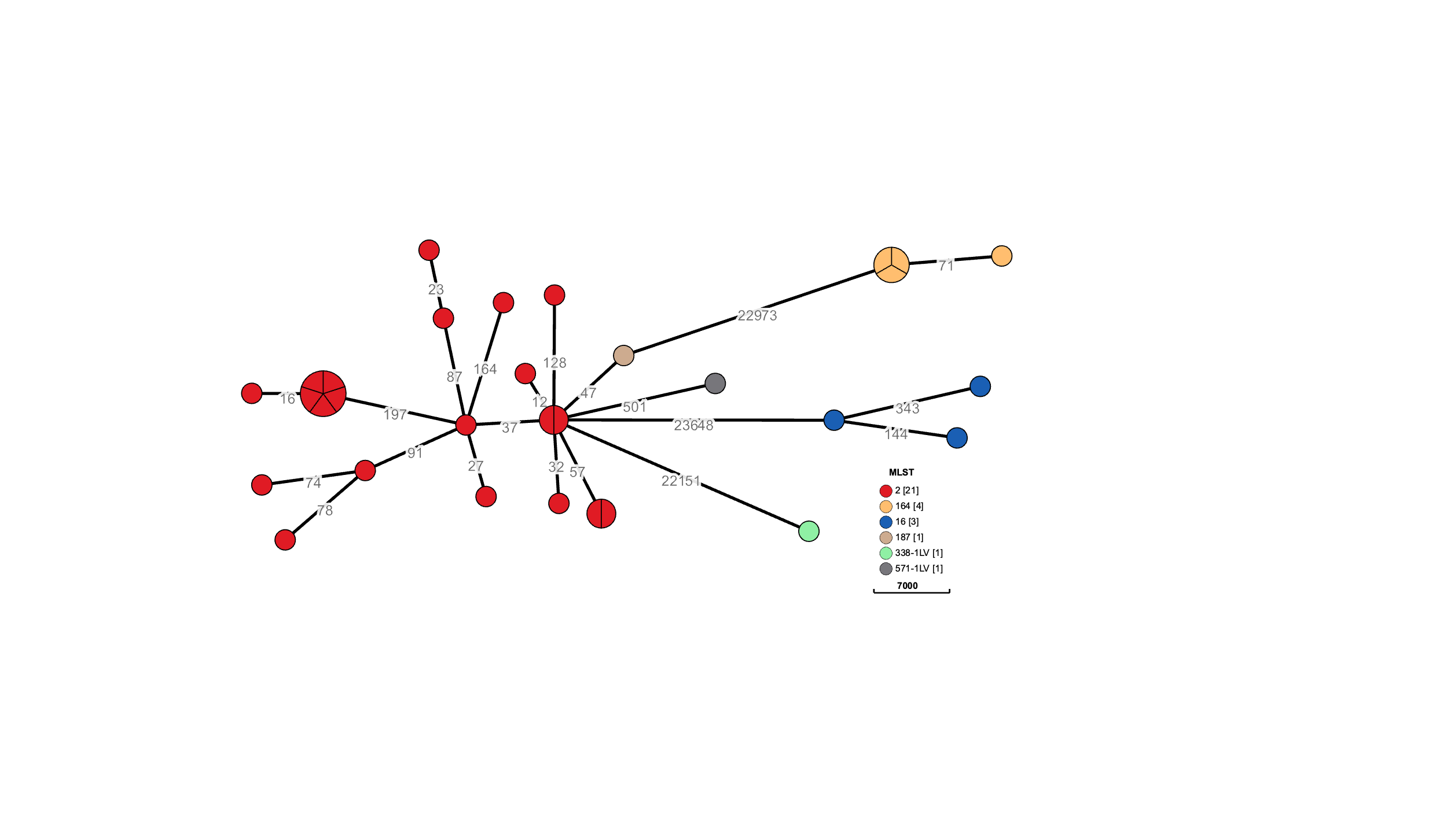


**Supplementary figure 2. Co-phylogeny of the genes *pirA* and *piuA* related to cefiderocol resistance in *A. baumanii* isolates.** Phylogenetic tree was obtained using the alignment of the predicted amino acid sequence of both genes. Paired are matched with a color-coded line indicating the resistance status to cefiderocol. Green lines indicate sensitive isolate with a MIC value < 1µg/mL, orange lines are isolates with a MIC value of 1 µg/mL (elevated MIC which may result in impaired clinical response) while the resistant isolates with MIC values >2 mg/L and/or zone diameter <17 mm are depicted with a red line. MLST was defined by the Pasteur scheme. Shimodaira–Hasegawa test was used to alternate the topologies of the whole sequences. The scale length (range: 0–1) represents the difference between sequences (range: 0–100%).

**
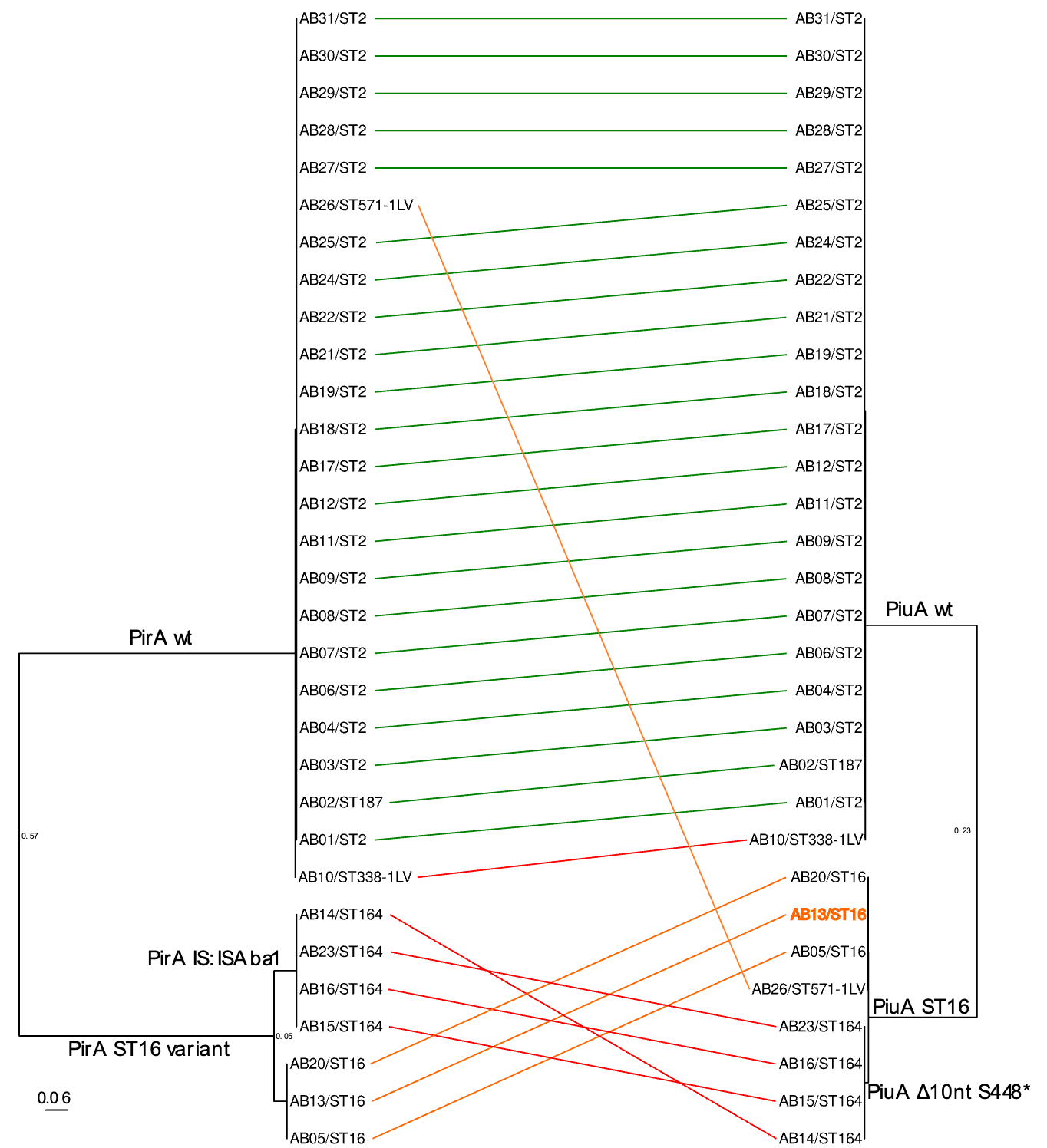
**
